# Veno-venous versus Veno-arterial extracorporeal membrane oxygenation for the management of primary graft dysfunction following lung transplantation: A systematic review and meta-analysis

**DOI:** 10.1016/j.jhlto.2025.100437

**Published:** 2025-11-14

**Authors:** Erlon de Avila Carvalho, Bruna Carregal Coelho, Rachid Eduardo Noleto da Nobrega Oliveira, Lucas Monteiro Delgado, Felipe S. Passos, Tulio Caldonazo, Marcelo Cypel

**Affiliations:** a Hospital das Clínicas, Federal University of Minas Gerais, Belo Horizonte, Minas Gerais, Brazil; b Federal University of Ouro Preto, Ouro Preto, Minas Gerais, Brazil; c Department of Thoracic Surgery, Barretos Cancer Hospital, Barretos, Brazil; d Department of Thoracic Surgery, MaterDei Hospital, Salvador, Brazil; e Department of Cardiothoracic Surgery, Jena University Hospital, Jena, Germany; f Division of Thoracic Surgery, Toronto Lung Transplant Program, Toronto General Hospital, University Health Network, University of Toronto, Toronto, Canada

**Keywords:** Lung transplantation, Primary graft dysfunction, Extracorporeal circulation membrane oxygenation

## Abstract

**Background:**

Primary graft dysfunction (PGD) is the leading cause of early morbidity and mortality after lung transplantation. In its severe forms, extracorporeal membrane oxygenation (ECMO) may be required. However, there is no consensus on whether venovenous (VV) or venoarterial (VA) ECMO modality provides superior outcomes. The objective of this meta-analysis is to compare the use of VV-ECMO versus VA-ECMO in the management of grade 3 PGD after lung transplantation.

**Methods:**

Three databases were assessed. Primary outcome was overall survival (OS). Secondary outcomes included cerebrovascular complications, incidence of pneumonia, reintubation, acute kidney injury, ICU length of stay and ECMO duration. Hazard ratios (HR), odds ratios (OR) and mean difference (MD) with 95% confidence intervals (CIs) were calculated. Reconstruction of time-to-event data and sensitivity analyses were performed for the primary endpoint.

**Results:**

Four studies including 210 patients (VV-ECMO: 142; VA-ECMO: 68) were analyzed. VV-ECMO was associated with improved early survival (HR 0.41; 95%CI 0.22 to 0.76; p=0.005) within the first 2 months after transplantation, although no significant difference was observed in long-term survival (HR 0.72; 95%CI 0.46 to 1.15; p=0.168). Cerebrovascular complications occurred significantly more often in the VA-ECMO group (OR 18.33; 95%CI 3.62 to 92.81; p=0.0004), while no significant differences were found in pneumonia, reintubation, acute renal failure, ICU length of stay, or ECMO duration.

**Conclusion:**

VV-ECMO appears to be associated with better short-term survival and fewer neurological complications compared to VA-ECMO in patients with grade 3 PGD after lung transplantation.

## Introduction

Primary graft dysfunction (PGD) is an acute lung injury syndrome that occurs within the first 72 h after lung transplantation, characterized by bilateral pulmonary infiltrates and hypoxemia. It represents the leading cause of early mortality in the immediate postoperative period, with a significant impact on long-term survival and quality of life of recipients. The incidence of grade 3 PGD at a single time point has been reported to be between 7.9% and 25.0%, whereas the incidence of at any time point during the first 3 days is approximately 30%.^1^, ^2^ Severe PGD is responsible for approximately 30% of patient mortality in the first month and 50% in the first year after transplantation.^3^, ^4^

In the most severe cases, conventional ventilatory support has proven inefficient, and extracorporeal membrane oxygenation (ECMO) has been widely used as a support strategy in refractory PGD. The venovenous (VV) modality is generally preferred in cases of isolated respiratory failure, whereas the venoarterial (VA) modality is considered in situations involving hemodynamic instability or ventricular dysfunction. However, there is no clear consensus on which modality provides better clinical outcomes in this population.^5^, ^6^

Despite the increasing use of ECMO in patients with PGD, comparative evidence between VV-ECMO and VA-ECMO modalities remain limited and heterogeneous. Individual studies have reported conflicting results regarding mortality, duration of support, and associated complications. The absence of randomized clinical trials and robust evidence-based guidelines underscores the need for a systematic synthesis of literature. Therefore, the objective of this meta-analysis was to compare outcomes of VV-ECMO versus VA-ECMO in the management of PGD after lung transplantation.

## Methods

This systematic review and meta-analysis followed the Cochrane Handbook for Systematic Reviews of Interventions and the Preferred Reporting Items for Systematic Reviews and Meta-Analysis (PRISMA) guidelines.^7^, ^8^ The study protocol was registered in the International Prospective Register of Systematic Reviews (PROSPERO, CRD420251116412).^9^

### Search strategy

We systematically searched PubMed, Embase, and Cochrane Library databases from inception to July 2025. We also searched the references of the included studies and previous systematic reviews and meta-analyses aiming for the inclusion of additional studies.^10^ The search strategy for each database is detailed in Supplemental Table S1.

### Study selection

Two independent authors (E.A.C. and B.C.C.) initially imported the studies into Rayyan Software (Qatar Computing Research Institute, Qatar Foundation) for deduplication and screening. Discrepancies between reviewers were resolved through discussion and consensus. Titles and abstracts were reviewed against pre-defined inclusion and exclusion criteria.

### Eligibility criteria

Inclusion in this meta-analysis was restricted to studies that met all of the following eligibility criteria: (1) randomized controlled trials (RCTs) or observational studies enrolling adult patients undergoing lung transplantation who developed grade 3 PGD; (2) studies comparing VV-ECMO versus VA-ECMO as treatment of grade 3 PGD; and (3) reporting at least one relevant clinical outcome. Exclusion criteria included studies involving pediatric patients, studies without clear separation between VV-ECMO and VA-ECMO, studies involving animal models, conference abstracts, case reports, studies with insufficient data and non-comparative study designs.

### Data extraction

Two authors (L.M.D. and F.S.P.) independently extracted data using a standardized form. The extracted variables included study characteristics (author, publication year, time frame, country, sample size, intervention and control groups) as well as patient demographics (age, sex, underlying disease, type of transplant, and reported outcomes).

### Risk of bias assessment

Two independent reviewers (F.S.P. and R.E.N.N.O.) assessed the Risk of Bias In Non-Randomized Studies of Interventions (ROBINS-I V2) tool.^11^ Disagreements were resolved through consensus. Publication bias was assessed only by visual inspection of the funnel plot, as formal statistical tests such as Egger’s test are recommended only when at least 10 studies are available.^12^

### Outcomes and subgroups analyses

The outcomes of interest were: (1) overall survival (OS); (2) cerebrovascular complications; (3) pneumonia; (4) reintubation; (5) acute renal failure; (6) ICU length of stay; and (7) ECMO support duration.

### Statistical analysis

Mean differences (MD) and odds ratios (ORs) with their 95% confidence intervals (CIs) were pooled for continuous and binary outcomes, respectively. Time-to-event data strategy was utilized for overall survival. A p-value < 0.05 was considered statistically significant for overall effect estimates. DerSimonian and Laird random-effects models were used for all outcomes.^13^ Heterogeneity was assessed using Cochran Q test and I² statistics; *p*<0.10 and I²>25% were considered significant for heterogeneity. A leave-one-out sensitivity analysis was performed for the primary endpoint to assess the impact of individual studies on the overall results, thereby ensuring the robustness of the findings. The Cochrane Handbook for Systematic Reviews of Interventions was used for data handling and conversion.^7^ All statistical analyses were conducted using R statistical software (version 4.4.2; R Foundation for Statistical Computing, Vienna, Austria).

### Individual patient survival data meta-analysis

We used the methods described by Guyot et al. to reconstruct individual patient data (IPD) from the Kaplan-Meier curves of all eligible studies for overall survival outcome.^14^ All Kaplan–Meier survival curves were pre-processed and digitized using WebPlotDigitizer, so that the values reflecting to specific timepoints with their corresponding survival information could be extracted. When available, numbers at risk were also collected.

The Kaplan–Meier method^15^ was used to calculate the overall survival rates. The Cox proportional hazards regression model was used to assess between-group differences. For these Cox models, the proportional hazards assumption was verified by plotting scaled Schoenfeld residuals, log–log survival plots, and predicted versus observed survival functions. We plotted survival curves using the Kaplan–Meier product limit method and calculated the Hazard Ratios (HRs) and 95% CIs of each group.

## Results

### Study and patient characteristics

**Figure 1:**
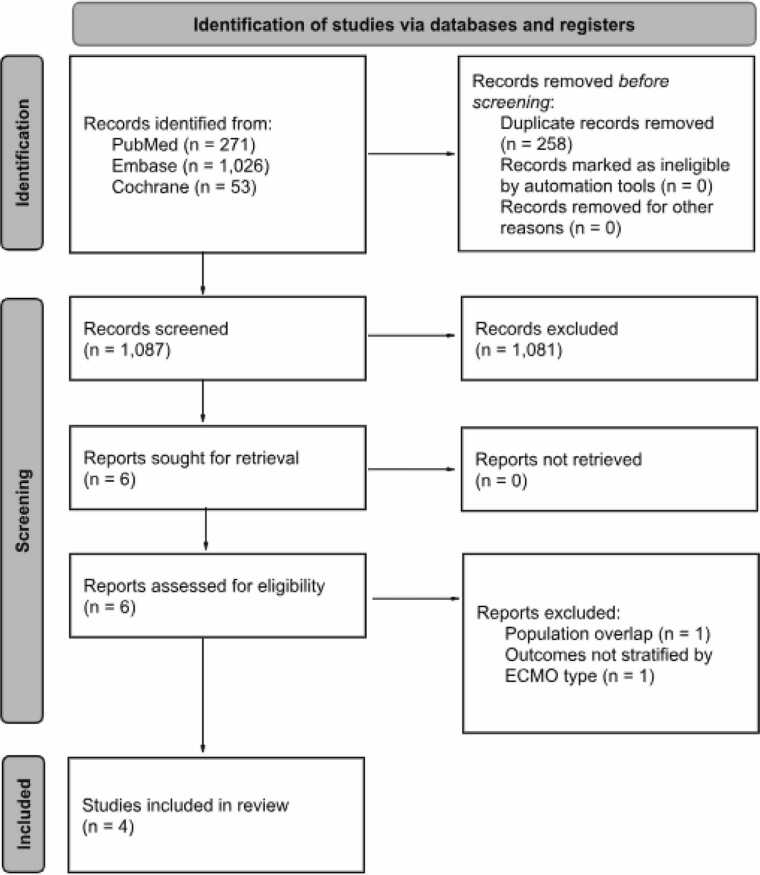
Preferred Reporting Items for Systematic Reviews and Meta-Analyses (PRISMA) flow diagram.

As detailed in Figure 1, the initial search yielded 1350 results. After deduplication, 1087 records were screened by title and abstract, resulting in 6 full-text articles assessed for eligibility. Of these, 4 met the inclusion criteria for inclusion in the final analysis, encompassing 210 patients.^16^, ^17^, ^18^, ^19^ Among these, 68 (32.38%) patients were submitted to VA-ECMO and 142 (67.62%) to VV-ECMO as a treatment strategy for grade 3 PGD. The main characteristics of the included studies are summarized in Table 1. Supplemental Table S2 lists the excluded studies from the full-text screening stage and the reasons for their exclusion.

**Table 1:** Baseline Characteristics of Included Studies

First author, Year	Country	Design	Time Frame	ECMO Patients, n (%)		Age, Years		Female, n (%)		Underlying Disease, n (%)				Single LT, n (%)		Bilateral LT, n (%)	
				VA	VV	VA	VV	VA	VV	ILD/IPF	COPD	CF	PVD	VA	VV	VA	VV
Bermudez, 2009	USA, Single-center	R-Obs	1991–2006	26 (45)	32 (55)	43*	50*	13 (50)	20 (62.5)	10 (17.2)	26 (44.8)	11 (19)	6 (10.3)	11 (42.3)	13 (40.6)	15 (57.7)	17 (59.4)
Hartwig, 2005	USA, Single-center	R-Obs	1992- 2005	15 (65.2)	8 (34.8)	41.9§	46.7§	7 (43.7)	5 (62.5)	6 (25)	3 (12.5)	2 (8.3)	5 (20.8)	2 (12.5)	0 (0)	14 (87.5)	8 (100)
Noda, 2024	USA, Single-center	R-Obs	2011–2022	14 (15.4)	77 (84.6)	NA		NA		NA		NA		0 (0)	0 (0)	14 (100)	77 (100)
Takahashi, 2023	USA, Single-center	R-Obs	2010–2020	13 (34.2)	25 (65.8)	52§	54§	4 (31)	15 (60)	21 (55)	10 (26)	3 (8)	3 (8)	1 (4)	0 (0)	24 (96)	13 (100)

CF: Cystic Fibrosis; COPD: Chronic Obstructive Pulmonary Disease; ILD: Interstitial Lung Disease; IPF: Idiopathic Pulmonary Fibrosis; LT: Lung Transplantation; NA: Not Available; PVD: Pulmonary Vascular Disease; R-Obs: Retrospective Observational; VA: Venoarterial; VV: Venovenous.

*median; §mean; †data from both groups combined.

### Outcomes

#### Overall survival

**Figure 2:**
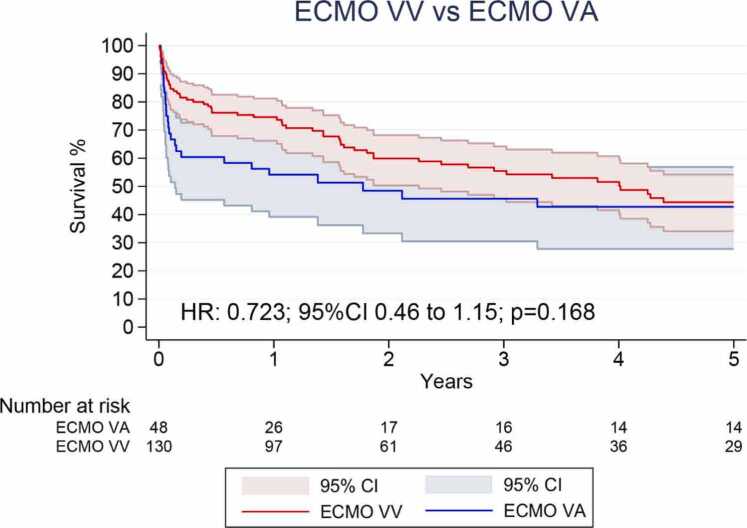
Overall survival for patients with grade 3PGD supported with VV-ECMO and VA-ECMO after lung transplantation. CI: Confidence Interval; ECMO: Extracorporeal Membrane Oxygenation; MH: Mantel–Haenszel; OR: Odds Ratio; VA: Venoarterial; VV: Venovenous.

Table 2 summarizes the meta-analysis findings. Overall, three Kaplan-Meier curves were digitized, processed and reconstructed. As shown in Figure 2, there was no significant difference regarding OS between VV-ECMO compared to VA-ECMO (HR 0.723; 95%CI 0.46 to 1.15; p = 0.168; Figure 2).

**Table 2:** Summary of Outcomes

Outcome	Number of Studies	Number of Patients	Effect Estimate, Random Model (95% CI, p-value)
Overall survival	3	187	HR 0.723; 95%CI 0.46 to 1.15; p = 0.168
Cerebrovascular events	3	152	OR 18.33; 95% CI 3.62 to 92.81; p=0.0004
Pneumonia	2	129	OR 1.33; 95% CI 0.50 to 3.54; p=0.5627
Reintubation	2	129	OR 0.99; 95% CI 0.23 to 4.27; p=0.9865
Acute renal failure	3	152	OR 1.76; 95% CI 0.65 to 4.71; p=0.2636
ICU length of stay	2	129	MD −1.90; 95% CI −8.12 to 4.31; p=0.5483
ECMO support duration	3	172	MD −0.42; 95% CI −1.08 to 0.23; p=0.2072

CI: confidence interval; ECMO: Extracorporeal membrane oxygenation; HR: Hazard ratio; ICU: Intensive care unit; MD: Mean difference; OR: Odds ratio.

#### Sensitivity analyses

**Figure 3:**
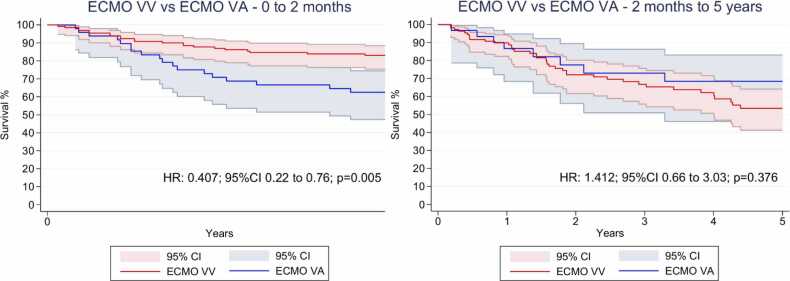
Landmark analysis for overall survival for patients with grade 3PGD supported with VV-ECMO and VA-ECMO after lung transplantation. CI= confidence interval, HR= hazard ratio; ECMO = Extracorporeal membrane oxygenation; VA: Venoarterial; VV: Venovenous.

Significant violations of the proportional hazards assumption were identified in the analysis of overall survival (Supplemental Figure S1). To address this, separate Cox models were performed for two distinct time intervals: 0 to 2 months and 2 months to 5 years post-transplant. In the early postoperative period (0–2 months), VV-ECMO support was significantly associated with improved survival (HR 0.407; 95%CI 0.22 to 0.76; p=0.005; Figure 3). However, this association did not persist beyond 2 months, as no significant difference was observed in the later period (p = 0.376; Figure 3).

The same tendency was detected when addressing the individual HRs of the studies in the two-stage meta-analysis using a random-effects model, which showed no significant difference in survival between VV-ECMO and VA-ECMO (HR 0.86; 95%CI 0.36 to 2.08; p = 0.74; I² = 60.3%; Supplemental Figure S2). The leave-one-out sensitivity analysis demonstrated that the survival benefit of VV-ECMO lost statistical significance whenever any single study was excluded, highlighting the fragility of this effect (Supplemental Figure S3). Finally, visual inspection of the funnel plot showed no relevant asymmetry, suggesting no major publication bias (Supplemental Figure S4).

### Clinical outcomes

**Figure 4:**
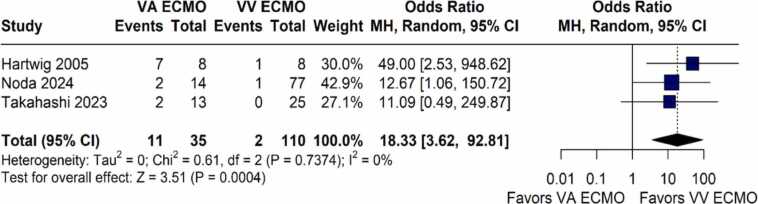
Forest plot comparing the occurrence rates of cerebrovascular events between patients with grade 3PGD supported with VV-ECMO and VA-ECMO in lung transplantation. CI: Confidence Interval; ECMO: Extracorporeal Membrane Oxygenation; MH: Mantel–Haenszel; OR: Odds Ratio; VA: Venoarterial; VV: Venovenous.

Figure 4 showed that the occurrence of cerebrovascular events was significantly higher in the VA-ECMO group compared to the VV-ECMO group (OR 18.33; 95%CI 3.62 to 92.81; I² = 0%; p = 0.0004; Figure 4).

**Figure 5:**
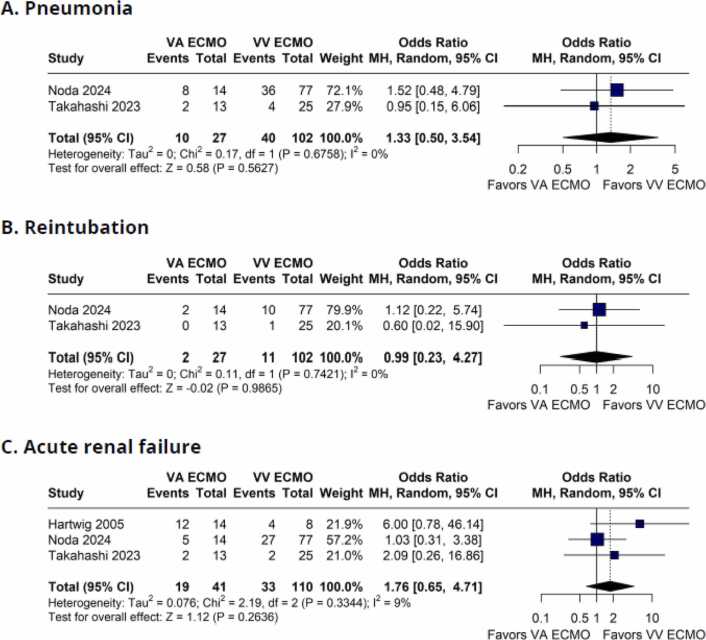
Forest plot comparing clinical outcomes between patients with grade 3PGD supported with VV-ECMO and VA-ECMO in lung transplantation. (A) Pneumonia; (B) Reintubation; and (C) Acute renal failure. CI: Confidence Interval; ECMO: Extracorporeal Membrane Oxygenation; MH: Mantel–Haenszel; OR: Odds Ratio; VA: Venoarterial; VV: Venovenous.

Figure 5 shows the forest plot for some clinical outcomes. There were no significant difference between groups for pneumonia (OR 1.33; 95%CI 0.50 to 3.54; I² = 0%; p = 0.5627; Figure 5A), reintubation (OR 0.99; 95%CI 0.23 to 4.27; I² = 0%; p = 0.9865; Figure 5B), and acute renal failure (OR 1.76; 95%CI 0.65 to 4.71; I² = 9%; p = 0.2636; Figure 5C).

**Figure 6:**
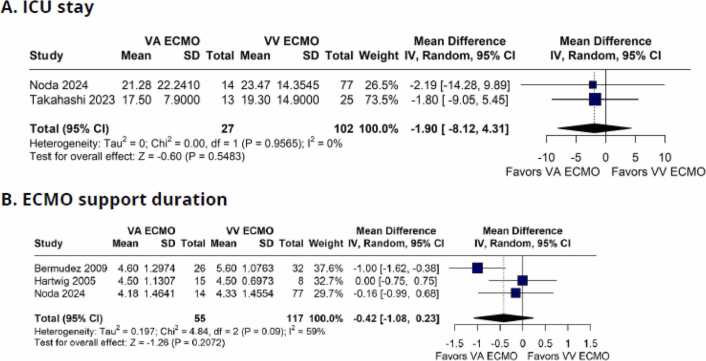
Forest plot comparing outcomes between patients with grade 3PGD supported with VV-ECMO and VA-ECMO in lung transplantation. (A) ICU length of stay, and (B) ECMO support duration. CI: Confidence Interval; ECMO: Extracorporeal Membrane Oxygenation; MH: Mantel–Haenszel; OR: Odds Ratio; VA: Venoarterial; VV: Venovenous.

In addition, there was no significant difference between groups for ICU length of stay (MD −1.90 days; 95%CI −8.12 to 4.31; I² = 0%; p = 0.5483; Figure 6A) and ECMO support duration (MD −0.42 days; 95%CI −1.08 to 0.23; I² = 59%; p = 0.2072; Figure 6B).

### Risk of bias assessment

Supplemental Figure S5 summarizes the risk of bias assessment. Across the included studies, the overall risk of bias was serious to critical, mainly due to confounding by indication. VV-ECMO was typically used for isolated hypoxemia, while VA-ECMO for hemodynamic instability or right ventricular dysfunction, creating baseline differences. The earliest studies had critical risk from significant confounding,^16^, ^17^ the absence of statistical adjustment, and era effects, while the more recent ones had serious risk due to persistent indication bias and lack of multivariable adjustment.^18^, ^19^ Other domains showed low risk, but all studies were non-randomized, limiting the ability to fully control baseline differences.

## Discussion

In this systematic review and meta-analysis of four studies including 210 patients, we comprehensively analyzed outcomes of VV-ECMO compared to VA-ECMO in lung transplant recipients with grade 3 PGD. Our main findings were as follows: (1) there was no significant difference in overall survival between VV-ECMO and VA-ECMO when analyzing pooled reconstructed curves; (2) sensitivity analyses demonstrated significant violations of the proportional hazards assumption, with VV-ECMO associated with improved survival during the early postoperative period (0–2 months), but this benefit was not sustained beyond two months; (3) cerebrovascular events were significantly more frequent in the VA-ECMO group, while no differences were observed for pneumonia, reintubation, or acute renal failure; and (4) ICU length of stay and ECMO support duration were comparable between groups.

In our study, patients supported with VV-ECMO demonstrated improved early survival compared with those managed with VA-ECMO. This benefit was limited to the first two post-operative months and was no longer significant beyond that period. This temporal pattern likely reflects the hemodynamic burden and embolic risks inherent to VA-ECMO, as well as the greater baseline severity of patients in whom it is typically employed, such as those with hemodynamic instability or right ventricular dysfunction.^19^, ^20^ Beyond this window, however, survival outcomes between VV-ECMO and VA-ECMO appeared to converge, suggesting that long-term prognosis may be more influenced by baseline disease severity and post-transplant recovery than by ECMO modality itself. This early survival gap raises the hypothesis that ECMO configuration may influence short-term outcomes in PGD3, particularly in patients without overt hemodynamic compromise. While causality cannot be established due to the observational nature of the included studies and the serious to critical risk of bias, these findings suggest that VV-ECMO may be a preferable first-line support strategy in selected patients, warranting further investigation through controlled prospective studies.

Previous work has shown that outcomes after lung transplantation with ECMO support are strongly influenced by patient selection and institutional strategies.^21^, ^22^ For example, Hoetznecker et al. reported exceptionally low PGD3 rates (1.3% at 72 h) using a highly standardized protocol of preemptive ECMO and controlled reperfusion, achieving excellent short- and mid-term survival.^23^ These results contrast with other cohorts where ECMO, and particularly VA-ECMO, is predominantly reserved for hemodynamically unstable patients or those with right ventricular dysfunction, populations at inherently higher risk of adverse outcomes.^24^ In this context, our finding of increased cerebrovascular events and worse early survival in VA-ECMO compared with VV-ECMO likely reflects both the higher baseline severity of patients undergoing VA-ECMO and the procedural risks associated with arterial cannulation.^25^ Taken together, these observations underscore the importance of rigorous neurological and vascular monitoring, as well as careful patient selection when determining the most appropriate ECMO modality. Given the well-established lower risk of thromboembolic and vascular complications associated with VV-ECMO, these findings further support its preferential use in selected cases of isolated respiratory failure.

While our findings support a short-term survival benefit and reduced risk of cerebrovascular complications with VV-ECMO, several important clinical questions remain. It is still uncertain whether VV-ECMO should be favored in all PGD3 cases without overt cardiac dysfunction, or whether specific PGD phenotypes, such as those with right ventricular failure or pulmonary hypertension, might benefit more from VA-ECMO despite its inherent risks. The absence of detailed hemodynamic or phenotypic stratification in the included studies limits definitive conclusions. Nonetheless, these results raise the hypothesis that ECMO configuration may influence early outcomes in selected subgroups of PGD3, reinforcing the need for prospective research tailored to individual patient characteristics.

The interpretation of PGD data in our analysis must also consider the evolution of diagnostic criteria over time. The 2005 ISHLT consensus first established standardized definitions and grading of PGD based on radiographic findings and PaO₂/FiO₂ thresholds at defined postoperative intervals.^26^ The 2016 update further refined these criteria, incorporating scenarios such as patients supported with ECMO or high-flow oxygen and standardizing the timing of assessments relative to reperfusion.^27^ While these revisions improved diagnostic uniformity, they may also contribute to heterogeneity in reported PGD rates, particularly when comparing older studies to more contemporary cohorts. Importantly, our meta-analysis included studies conducted under both the 2005 and 2016 definitions, which may partially explain variability in PGD incidence and outcomes across different eras and ECMO modalities.

No significant differences were observed in rates of pneumonia, reintubation, or acute renal failure, suggesting that these complications are less dependent on ECMO modality and more closely related to overall postoperative care and patient comorbidities. Similarly, ICU length of stay and ECMO support duration were comparable between VV-ECMO and VA-ECMO, highlighting that beyond the acute phase, both modalities perform similarly when appropriately indicated.

Future research should focus on the long-term outcomes associated with different ECMO modalities in the setting of PGD, including functional recovery, quality of life, and the burden of chronic complications. Standardizing protocols for VA- and VV-ECMO use, along with refining patient selection criteria, will be critical to optimizing support strategies.

### Study strengths and limitations

This meta-analysis is the first to use reconstructed time-to-event data to directly compare VV-ECMO and VA-ECMO in patients with grade 3 PGD after lung transplantation. A key strength of this approach is the ability to assess temporal effects on survival, revealing an early advantage for VV-ECMO that was not evident in crude pooled analyses.

Nonetheless, several limitations must be acknowledged. All included studies were observational, introducing inherent risks of treatment allocation bias. VA-ECMO was predominantly employed in patients with hemodynamic instability or right ventricular dysfunction, while VV-ECMO was typically reserved for isolated hypoxemia. These baseline differences generated confounding by indication, classified as serious to critical across studies, and complicate direct comparisons of outcomes. In addition, small sample sizes, variability in study eras, and heterogeneous institutional protocols further limit the generalizability of results.

Future multicenter prospective studies with standardized ECMO strategies are needed to validate these findings, reduce bias, and establish evidence-based guidelines for modality selection in the management of severe PGD.

## Conclusion

VV-ECMO in severe PGD after lung transplantation may be associated with improved early survival and a reduced risk of cerebrovascular complications compared to VA-ECMO, although these advantages diminish beyond the early postoperative period. These findings should be interpreted as hypothesis-generating, as they likely reflect a combination of patient selection factors and the procedure-related risks inherent to VA-ECMO.

## Data Availability Statement

The data underlying this article are available in the article and in its online supplemental material.

## Funding

This work was supported by the Deutsche Forschungsgemeinschaft (DFG, German Research Foundation to Tulio Caldonazo) Clinician Scientist Program OrganAge funding number 413668513, by the Deutsche Herzstiftung (DHS, German Heart Foundation to Tulio Caldonazo) funding number S/03/23 and by the Interdisciplinary Center of Clinical Research of the Medical Faculty Jena.

## Disclosures

None.

## Declaration of Competing Interest

The authors declare that they have no known competing financial interests or personal relationships that could have appeared to influence the work reported in this paper.

## Footnotes

Appendix A Supplemental data associated with this article can be found in the online version at doi:10.1016/j.jhlto.2025.100437.
