# Supplementary material for: Veno-venous versus Veno-arterial extracorporeal membrane oxygenation for the management of primary graft dysfunction following lung transplantation: A systematic review and meta-analysis
Source: JHLT Open. 2025 Nov 14;11:100437. doi: 10.1016/j.jhlto.2025.100437 (PMC12721064; doi:10.1016/j.jhlto.2025.100437)

**SUPPLEMENTARY TABLES AND FIGURES LEGENDS:**

**Supplementary Table S1.** Search strategy.

**Supplementary Table S2.** Reasons for exclusion of studies after full-text eligibility assessment.

**Supplementary Figure S1**. Plot for the test of Proportional Hazards Assumption for the primary endpoint (Overall Survival).

**Supplementary Figure S2**. Two-stage meta-analysis for the primary endpoint (Overall Survival).

**Supplementary Figure S3.** Leave-one-out analysis for the primary endpoint (Overall Survival).

**Supplementary Figure S4.** Funnel plot for the primary endpoint (Overall Survival).

**Supplementary Figure S5.** Critical appraisal of studies according to Risk Of Bias In Non-randomized Studies of Interventions (ROBINS-I)

**Supplementary Table S1.** Search strategy.

| PubMed | "Lung Transplantation"[Mesh] OR "lung transplant"[Title/Abstract]) **AND** ("Primary Graft Dysfunction"[Mesh] OR "PGD" [Title/Abstract]) **AND** ("Extracorporeal Membrane Oxygenation"[Mesh] OR "ECMO"[Title/Abstract] OR "veno-venous"[Mesh] OR "VV ECMO"[Title/Abstract] OR "veno-arterial"[Mesh] OR "VA ECMO"[Title/Abstract]). |
| --- | --- |
| Embase | ("Lung Transplantation" OR "lung transplant") **AND** ("Primary Graft Dysfunction" OR "PGD") **AND** ("Extracorporeal Membrane Oxygenation" OR "ECMO" OR "veno-venous" OR "VV ECMO" OR "veno-arterial" OR "VA ECMO") |
| Cochrane | ("Lung Transplantation" OR "lung transplant") **AND** ("Primary Graft Dysfunction" OR "PGD") **AND** ("Extracorporeal Membrane Oxygenation" OR "ECMO" OR "veno-venous" OR "VV ECMO" OR "veno-arterial" OR "VA ECMO") |

**Supplementary Table S2.** Reasons for exclusion of studies after full-text eligibility assessment.

| First author, year [reference] | Reason for exclusion |
| --- | --- |
| Harano, 2021 [1] | Overlapping patient cohort with two other studies utilizing the database of University of Pittsburg Medical Center |
| Fisher, 2007 [2] | Outcomes not stratified by ECMO type. |

1-Harano T, Ryan JP, Morrell MR, Luketich JD, Sanchez PG. Extracorporeal Membrane Oxygenation for Primary Graft Dysfunction After Lung Transplantation. ASAIO J. 2021 Sep 1;67(9):1071-1078. doi: 10.1097/MAT.0000000000001350. PMID: 33470638.

2-Fischer S, Bohn D, Rycus P, Pierre AF, de Perrot M, Waddell TK, Keshavjee S. Extracorporeal membrane oxygenation for primary graft dysfunction after lung transplantation: analysis of the Extracorporeal Life Support Organization (ELSO) registry. J Heart Lung Transplant. 2007 May;26(5):472-7. doi: 10.1016/j.healun.2007.01.031. PMID: 17449416.

**Supplementary Figure S1**

**
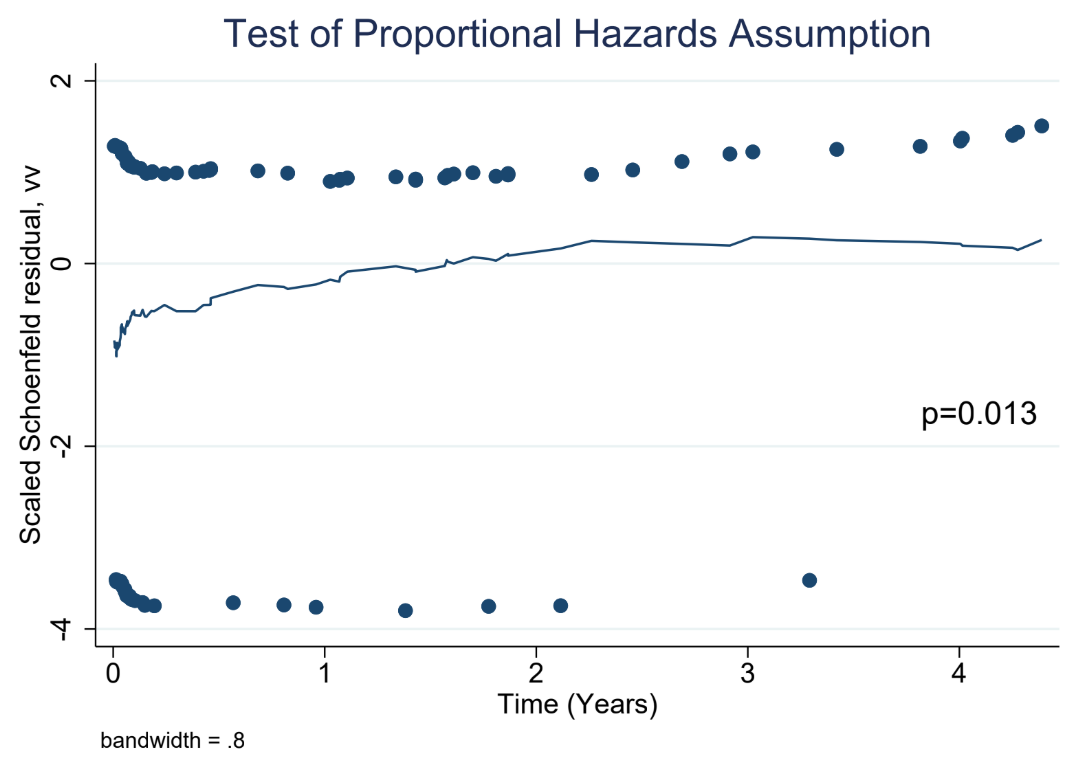
**

**Supplementary Figure S2**


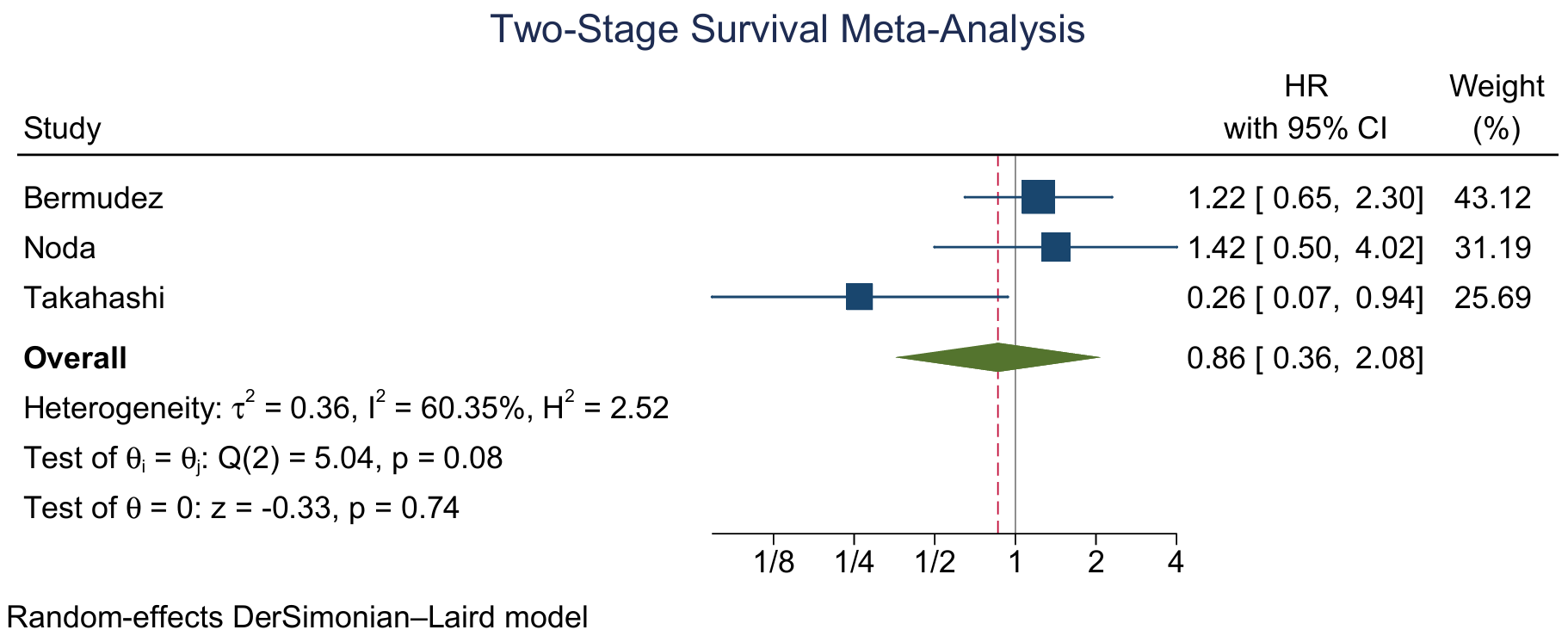


**Supplementary Figure S3**


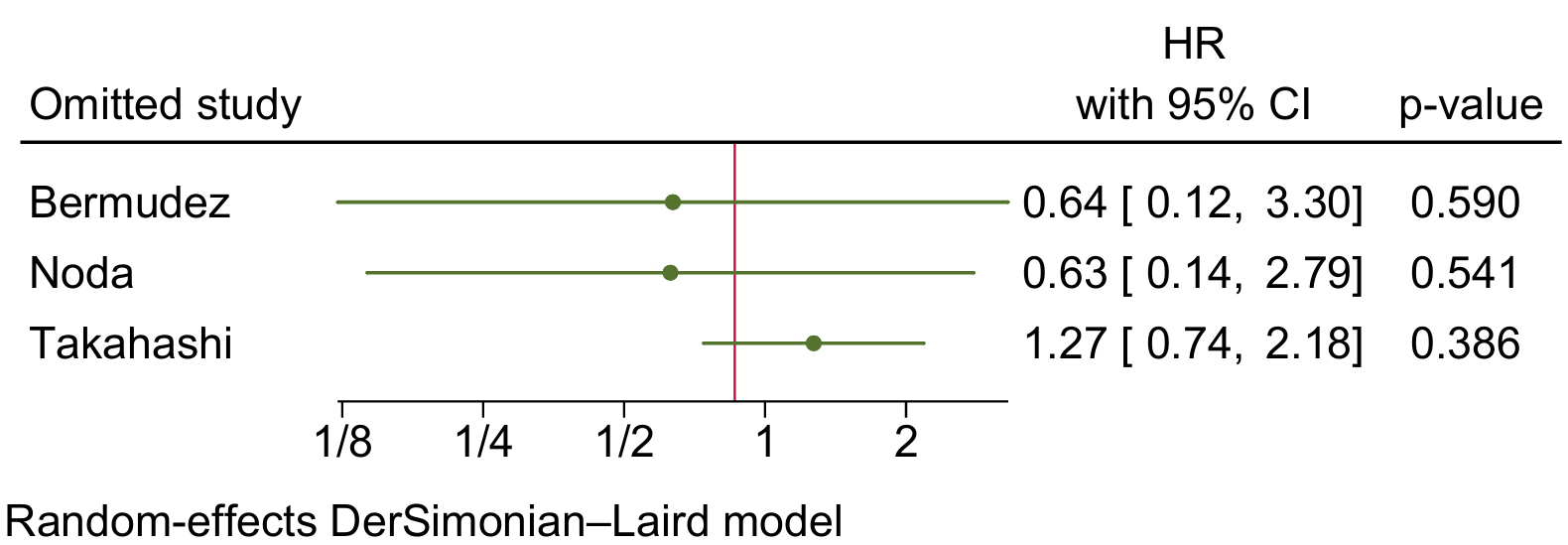


**Supplementary Figure S4**


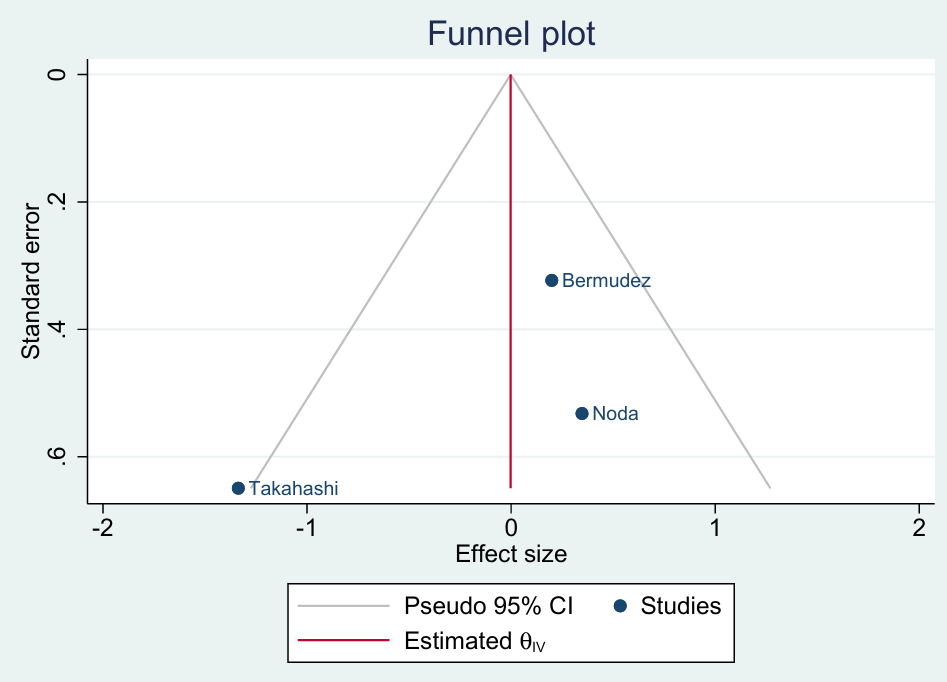


**Supplementary Figure S5**


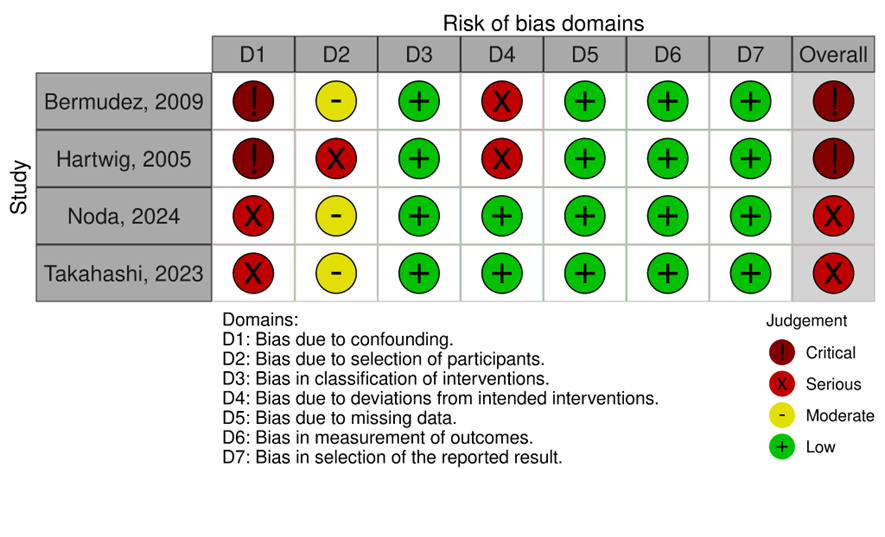

Supplement: Supplementary file 1 — Supplemental material [file mmc1.docx]
